# Prevalance of Celiac Disease in Patients with Inflammatory Bowel Disease in Turkish Population

**DOI:** 10.1155/2019/6272098

**Published:** 2019-12-11

**Authors:** Göksel Bengi, Musa Cıvak, Mesut Akarsu, Müjde Soytürk, Ender Ellidokuz, Ömer Topalak, Hale Akpınar

**Affiliations:** ^1^Gastroenterology Unit, Department of Internal Medicine, Dokuz Eylül University, İzmir, Turkey; ^2^Department of Internal Medicine, Dokuz Eylül University, İzmir, Turkey

## Abstract

**Background:**

Celiac disease (CD) and inflammatory bowel disease (IBD) involve inflammation of the gastrointestinal lumen, which environmental, genetic, and immunological factors have a role in their pathogenesis. The prevalence of celiac disease in IBD ranges from 0% to 14%. In this study, our aim was to determine the prevalence of CD in IBD patients followed by us who are attending the hospital or outpatient clinic over a period of time of seven years.

**Methods:**

Seven hundred and fifty nine patients (425 M, 334 F, mean age: 46.75, 396 ulcerative colitis (UC), 363 Crohn's disease (CrD)) diagnosed and followed up for IBD between January 2009 and July 2016 were evaluated retrospectively, and clinical, demographic, laboratory, and endoscopic data were collected.

**Results:**

CD was investigated in 79 (%10.4) inflammatory bowel disease patients according to symptoms, and in 5.06% (*n* = 4) of them, we diagnosed CD. The most common indication for investigating for CD was iron deficiency anemia unreponsive to iron supplementation.

**Conclusions:**

We did not find an increased prevalance of celiac disease in Turkish IBD patients in this study. In the presence of refractory iron deficiency anemia without any other cause in IBD patients, investigations for celiac disease should be considered.

## 1. Introduction

Celiac disease (CD), Crohn's disease (CrD), and ulcerative colitis (UC) are inflammatory diseases of the gastrointestinal lumen with environmental, genetic, and immunologic factors playing a role in their pathogenesis. CD is an immunologic disease that occurs as a result of gluten intake with foods in genetically susceptible individuals and results in malabsorption in the small intestine [1]. Although the prevalence of CD is reported to be 1% in the general population, its incidence is progressively increasing in developed countries. CD diagnosis should be confirmed on the basis of a compilation of findings obtained from medical history, physical examination, serologic tests, and upper gastrointestinal endoscopy accompanied with histologic analysis of multiple duodenal biopsies; improvement should be seen by avoiding gluten in diet [2].

CD can also have clinical signs apart from its classical signs (diarrhea, malnutrition, weight loss, steatorrhea, edema secondary to hypoalbuminemia, and delayed growth and development in children). CD can be one of the underlying causes of nonspecific symptoms like dyspepsia and reflux or in numerous broad-spectrum clinical pictures (iron deficiency anemia (IDA), elevation of liver enzymes, infertility, premature osteoporosis, type 1 diabetes mellitus (T1DM), neurologic symptoms, etc). Given its nonspecific presentation, CD could be underdiagnosed with the actual prevalence being three- to sevenfold higher than what is currently reported [3–7]. CD is an autoimmune disease and can be associated with other autoimmune diseases like T1DM, autoimmune thyroid diseases, Addison's disease, and primary biliary cirrhosis [8–13].

Both CD and inflammatory bowel disease (IBD) occur more frequently in individuals with genetic risk factors. Moreover, some autoantibodies are noted both in CD and IBD. For instance, anti-Saccharomyces cerevisiae antibodies (ASCAs) were positive in 39%-70% of patients in CrD [14]. ASCA positivity was reported at a rate of 67% in CD [15]. In a study by Snook et al. [16], antinuclear antibodies (ANAs) were positive at a rate of 25%-51% in UC and 8%-17% in CD. Moreover, a correlation was shown between disease activity and antitissue transglutaminase antibody (anti-TgA) positivity in CrD [17].

It is difficult to make the diagnosis of CD in patients previously diagnosed with IBD because symptoms of diarrhea, weight loss, and abdominal pain are common in both conditions and thus can be confusing. Biochemical changes would also be similar and therefore not very helpful in making the diagnosis. Comb tooth appearance in the duodenum which is one of the most characteristic endoscopic signs in the diagnosis of CD can also be present in CrD, and villous atrophy can even be seen in 20% of patients in CrD [18, 19].

Association of IBD with CD has recently been shown in case reports and case series but prevalence studies have contradictory results [18–28]. In the light of these literature data, the prevalence of CD in IBD varies in the range of 0% and 14%.

Screening for CD in those diagnosed with IBD is still controversial. In this study, we aimed to determine the prevalence of CD in IBD patients followed up by us who are attending the hospital or the outpatient clinic over a period of time of seven years.

## 2. Materials and Methods

759 patients (425 males, 334 females, mean age: 46.75) followed up for IBD (396 ulcerative colitis, 363 Crohn's disease) between January 2009 and July 2016 were evaluated retrospectively. Clinical, demographic, laboratory, and endoscopic data were collected for further analyses. The diagnosis of IBD was made with a combination of endoscopic, radiologic, and histopathologic criteria. The patients who are followed up with IBD diagnosis and for whom investigations directed at celiac disease were requested for various reasons (most common iron deficiency anemia that does not recover despite sufficient treatment received, unexplained elevations of liver enzymes, or persistence of malabsorption findings despite optimal treatment) were included in this study. Demographic and clinical information like age, gender, disease type and involvement, medical therapies received, presence or absence of anemia therapy, antibodies tested for the diagnosis of celiac disease, endoscopic findings if upper GI endoscopy was performed, and pathologic results were recorded. Involvement of the disease was determined according to endoscopic and radiologic findings.

Diagnosis of CD in registered patients was made with the presence of antiendomysium IgA (EMA) (Euroimmun, EUROPLUS liver (monkey) and gliadin (GAF-3X) antigen, IFAT method) and/or antitissue transglutaminase IgA antibodies (anti-TgA) (Euroimmun, with ELİSA method and negative: <20 RU/mL, positive: ≥20 RU/mL) and pathology results of endoscopic duodenal biopsies. In addition, it was also checked if patients were tested for IgA levels (normal reference range: 70-400 mg/dL) in order to exclude selective serum IgA deficiency in the patients. When histopathologic results were evaluated, patients were divided into groups according to the Marsh classification used in the diagnosis of CD [29]: Marsh type 1, infiltrative lesions with lymphocyte dominance in more than 30 of 100 epithelial cells; Marsh type 2, infiltrative/hyperplastic lesions; and Marsh type 3, intraepithelial lymphocytosis and crypt hyperplasia accompanied by villous atrophy ((a) partial villous atrophy, (b) subtotal villous atrophy, and (c) total villous atrophy). Particularly, villous atrophies in the duodenum noted in the pathology report like Giardia lamblia infection, tropical sprue, collagenous sprue, food protein hypersensitivity (cow's milk, eggs, fish, rice, and chicken), infiltrative diseases of the duodenum like duodenitis were excluded.

In addition, consent was obtained from the ethics committee for noninvasive clinical trials of Dokuz Eylul University Medical Faculty dating 16 March 2017 and with number 2017/05-29. The study was conducted according to the Declaration of Helsinki keeping patient information confidential.

Statistical analysis was performed using the SPSS 19.0 for Windows (SPSS, Inc.; Chicago, USA) package program. Descriptive values were given as number (*n*), percentage (%), mean, standard deviation (SD), and median. Pearson's chi-squared test was used to test for an association between two categorical variables. Continuous variables were compared with nonparametric tests (Mann–Whitney *U* test and Kruskal-Wallis test) because they did not have a normal distribution when evaluated for normality of distribution with Kolmogorov-Smirnov and Shapiro-Wilk tests. Level of statistical significance was *p* < 0.05.

## 3. Results

Demographic data, IBD treatments, and anemia profiles of the patients in the study has been shown in Table 1. Work-up for CD had been carried out in only 79 (10.4%) of 759 patients. The main reason for investigating for CD was recurrent IDA despite replacement therapy.

CD was investigated in 79 (%10.4) inflammatory bowel disease patients according to symptoms, and in 5.06% (*n* = 4) of them, we diagnosed CD.

CD was detected in 0.52% of all IBD patients in the follow-up program and in 5.06% (*n* = 4) of 79 inflammatory bowel disease patients investigated for CD.

**Figure 1:**
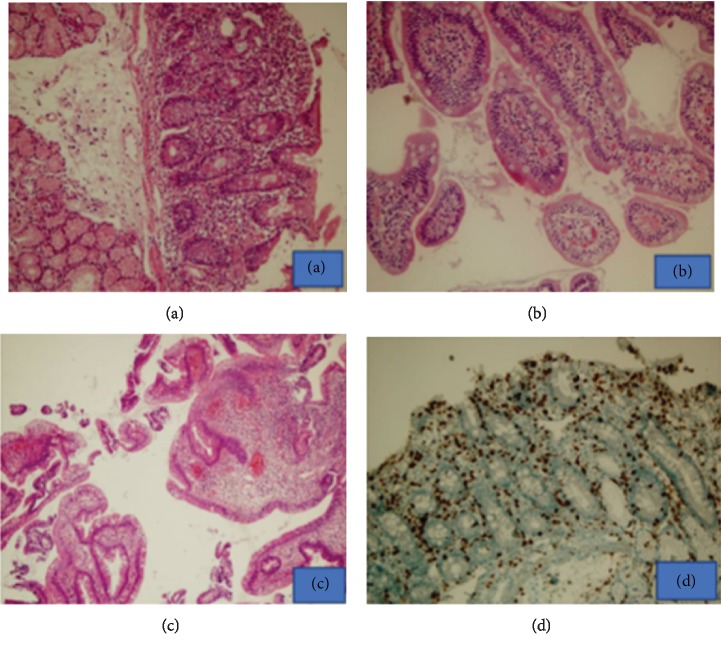
(a) Villous blunting in the small intestine mucosa (H&E, 200x). (b) Preserved villous setup and slightly increased intraepithelial lymphocytes (H&E, 400x). (c) Duodenum tissue with active duodenitis and gastric metaplasia (H&E, 40x). (d) Increase of CD3 and lamina propria and lymphocyte increase in epithelium (H&E, 200x).

Mean age of the patients found to have CD was 50.5 (±20.5), and all four were female. Antibodies were positive in the blood (*n* = 3, anti-tTG; *n* = 4, anti-EMA; *n* = 3, both anti-tTG and anti EMA) in the four cases found to have CD. Histopathologic examination of duodenal biopsy revealed changes consistent with Marsh type 2 (*n* = 1) and Marsh type 3 (*n* = 3) (Figure 1).

Of the CD patients, one had UC and three had CrD, and all four patients with associated IBD and CD had IDA. Both IDA and liver function tests revealed altered results in one of the patients.

The clinical characteristics of the patients with celiac disease are given in Table 2.

None of the patients with both IBD and CD had any of the major signs or symptoms suggesting malabsorption like amenorrhea, osteoporosis, low albumin, or cholesterol levels. These four patients who also had iron deficiency anemia were started on a gluten-free diet, and they were followed for 9, 11, 2, and 22 years, respectively. Anemia was not detected in the final follow-up. Despite a low-gluten diet, one patient's liver enzymes did not decrease, and NASH (nonalcholic steatohepatitis) was determined as the cause of high liver enzymes in the follow-up examinations. The patients were not followed endoscopically.

## 4. Discussion

Prevalence of CD is approximately 1%-2% in the general population [2]. Adults are usually diagnosed in the 4th and 5th decades of life, and there is a delay of 4.9-11 years in making the diagnosis [30]. Other autoimmune diseases are also common especially in celiac patients who have not been treated for a long time. The greatest predisposing factor in this clinical condition is the presence of the same HLA class II haplotype in different diseases [31]. Since the first demonstration of CD and CrD association in three different Sicilian families by Cottone and Capello in 1989 [32], this association has been reported in many case series. Although CD is seen in IBD patients, contrary to this, the prevalence of IBD was found to be increased five- to tenfold in CD compared with the general population [33]. However, in many cases, this association was coincidentally noted. It is difficult to make the diagnosis of CD in patients previously diagnosed with IBD, because symptoms of diarrhea, weight loss, and abdominal pain are common in both conditions and can be confusing. For example, many IBD patients have been diagnosed with CD when further investigations were made because of diarrhea that did not improve or IDA that persisted following antiinflammatory therapy [25–27]. Four of our IBD patients who were found to have CD had long-standing IDA. Although IDA is a commonly seen condition in IBD, it is emphasized that anemia should be absolutely treated in these patients [34]. CD should be kept in mind usually in unexplained or resistant IDA, and especially if anemia is mild and another malabsorption sign is not present, etiology of a long-standing anemia can be missed [35]. Possibility of CD should be kept in mind in the case of anemia unresponsive to iron therapy in IBD patients [36]. Nevertheless, our patients found to have CD were all female, and although involvement sites were different in all of them, a specific involvement pattern that would be a predisposing cause regarding CD was not observed.

The presence of villous atrophy in duodenal biopsies was first shown in 1965 by Salem in 20% of those with UC [37]. Following this, most of the publications showing the association of IBD with CD were case reports or small case series and prevalance studies are very few. Prevalence of CD in the IBD cohort groups in these studies varies between 0.3% and 14% [20, 25, 38, 39]. Our study is the first study in Turkey that investigates CD prevalence in IBD patients. We investigated CD at an approximate rate of 10% mainly because of recurrent IDA in the IBD patients in our follow-up. IDA was found in 45.3% (*n* = 341) of patients.

CD was found in 5.06% of the 79 patients investigated which is consistent with the rate reported in the literature. On the other hand, when all IBD patients were considered, CD prevalence was found to be 0.52% in our study. Consequently, our data do not support routine screening for CD in patients with clinically unequivocal CD. CD was seen more frequently in CrD (3/40, 7.5%) than in UC (1/39, 2.56%) (*p* = 0.317). Similarly, in the study by Tursi et al. [20], a high rate of assocition with CD was also shown in a small group of CrD patients. However, CD had a higher rate of association with UC in the study by Snook et al. [16], contrary to our study. Moreover, these authors also showed a high association of UC with other autoimmune diseases and attributed it to immunologic dysregulation. CrD is a multifactorial disease with environmental, genetic, and immunologic changes, whereas CD is characterized with hypersensitivity to gliadin. Association of CrD with CD can be explained with intraepithelial T cells which are the key points responsible for immunopathogenesis of both diseases. Human gastrointestinal system is a complex ecosystem with a balance between antigenic stimuli and immune response. It is characterized with increases in chronic *α* immunologic response. While an immunologic response with predominant T helper 1 pathway is predominant in both CD and CrD, cellular apoptosis is decreased and there is chronic inflammation especially in the lamina propria [40, 41]. In addition, caspase-8 is decreased and there is resistance against apoptosis in IL-15-mediated intraepithelial T cells. IL-15 expression is increased in both CrD and CD [42, 43]. Nevertheless, other cytokines like TNF-*α*, interferon-*γ*, and IL-8 playing a role in cell-mediated immunopathogenesis are also increased in these two diseases [44]. The question of why all CD and CrD are not associated can be answered with the close relation between CD and HLA DQ2 and HLA DQ8. However, CD can develop in CrD showing HLA DQ2 and DQ8 positivity [45].

Duodenal biopsy sampling plays a key role in the diagnosis of CD in that sometimes patients are mistakenly diagnosed with CrD. Severe villous atrophy and increased intraepithelial lymphocytosis were seen in CrD in a previous study, but serologic tests for CD were not performed in this study [19]. In our patient population, serologic tests were positive in all the patients found to have CD, and at the same time, their pathologic results were consistent with type 2 and 3 modified Marsh classification. In previous studies, human recombinant anti-tTG had a high rate of false positivity in connective tissue disorders and other autoimmune GIS disorders (except for PBS) [46]. Therefore, histologic results are also important in addition to EMA and anti-tTG positivity which are celiac antibodies when making the diagnosis of CD especially in IBD patients.

Although it is known in the literature that the risk of developing IBD during follow-up is increased in celiac disease patients, there is no increased risk for developing CD during follow-up in IBD patients. For example, Yang et al. [47] have conducted a study with the largest number of patients and have shown the presence of IBD in 27 of 455 patients (5.9%) with histologically proven CD (5 UC, 5 CrD, 17 microscopic colitis). In a large Swedish mortality study, mortality risk because IBD was found to be higher than bowel cancer or lymphoma in patients with CD (70.9, 95% CI, 36.6-123.9) [48]. Studies did not only show association of IBD with CD, but they also showed that the clinical picture of the patient is more serious and even a colectomy is needed in the case of association [47]. In our study, our patients with associated IBD-CD both responded to gluten-free diet and were in clinical remission regarding IBD.

The pathophysiology of the relationship between CD and IBD is still not clearly understood. While there is a close correlation between CD and HLA-DQ2 and HLA-DQ8 allelles, there is no such correlation in IBD [49]. However, CD developed in CrD carrying HLA-DQ2 or HLA-DQ8 alleles. Therefore, other potential genetic factors that would cause both diseases should be identified. Cottone and Capelllo [32] have found increased UC prevalence in first-degree relatives of celiac patients. On the other hand, Lopez-Vasquez et al. [50] have shown that major histocompatibility complex class I chain-related gene A (MICA) was expressed more from the gastrointestinal epithelium in CD with IBD transformation. MYO IXB gene known to be related with CD was shown to undergo mutation also in IBD patients in recent studies [51]. This gene contributes to the integrity of cellular skeleton, cell polarity, and tight junctions by encoding elements of myosin superfamily. As a result of mutation in this gene, mucosal intestinal barrier defects like increased permeability in tight junctions have been described in both CD [52, 53] and IBD [54]. Increased intestinal permeability leads to increase in antigen presentation as well as increase in the formation of autoantibodies and bacterial translocation, thus playing a role in IBD pathogenesis. As a result of increased intestinal permeability in CrD, many bacteria mimic 57-68 and 62-75 gliadin sequences and activate cytokine cascade (IL-15, IL-2, TNF-*α*, and IFN-*γ*), and it is hypothesized that this leads to Th1-mediated immune reaction and causes the development of CD. This hypothesis has recently been proved by the observation of seroreactivity to Saccharomyces cerevisiae not only in CrD but also in CD at a high rate [55]. Having both DQ2 and DQ8 alleles and increased gliadin presentation are not responsible alone in the pathogenesis of CD. This can explain to us why IBD is more common in CD while incidence of CD is not increased in IBD. Moreover, use of biologic agents can lead to the formation of autoantibodies and development of autoimmune diseases [56]. In contrast to the study by Leeds et al. [21] where antibody levels were higher in those taking infliximab than those not taking it, none of the patients found to have CD-IBD association in our study used anti-TNF. In addition, there was no siginificant difference among the CD antibody profiles of our two patients who were on AZA therapy.

Our study also had some limitations and controversial aspects. Only 10% of our IBD patients were screened for CD upon clinical suspicion. Indeed, only the prevalence 4/79 (5.06%) was really detected in the study, while that of 4/759 (0.52%) is a presumed finding that does not take into account the silent (asymptomatic) CD patients, as well as the mono/paucisymptomatic CD patients with a clinical picture partially or totally overlapping on that of IBD. As a consequence, the CD prevalence in the cohort of 759 IBD patients could be underestimated. With regards to isolated IgA deficiency, only 16 of 79 patients, whose celiac antibodies were measured, were tested for IgA levels. Although microscopic colitis is not accepted as IBD in general practice, it has been evaluated in the IBD subgroup in some studies [21, 47], so results can be different from our results because we only screened UC and CrD cases for CD prevalence in our study. HLA DQ2 and DQ8 status of our IBD patients found to have CD is not known. As we could not make a healthy evaluation of IBD disease activities because our study was a retrospective one, a correlation between IBD disease activity and celiac serologic marker levels could not be established. Nevertheless, since endoscopic follow-up of the patients could not be reached, histologic changes occurring as a result of gluten-free diet could not be observed.

In conclusion, our study is the first study that investigated CD prevalence in IBD patients in Turkey. In our study, CD was found in a very small portion of patients with asymptomatic IBD and in 5.06% of patients screened because of clinically suspicious CD, and prevalence was higher in CrD than in UC. Therefore, since according to our results, there was no significant evidence about increased risk of CD in patients diagnosed with IBD. However, the presence of CD should also be kept in mind in patients followed with the diagnosis of IBD and in the presence of resistant IDA, malabsorption signs, or in association with other autoimmune diseases, and further invastigations should be planned. Future studies should focus on the immunopathologic mechanisms like genetic markers and intestinal permeability that form the basis for the alliance between these two disease groups and on what can be done in preventing the development of the other clinical picture in the presence of IBD or CD.

## Figures and Tables

**Table 1:** Demographic data, IBD treatments, and anemia profiles of the patients in the study.

	CrD	UC	*p*
Gender (female/male) (*n*)	151/212	183/213	0.263^∗^
Mean age (years) (SD)	45.9 (±14)	47.5 (±16.1)	0.157^∗∗^
Mean duration of disease (years) (SD)	6.42 (±5.7)	8.29 (±6.8)	<0.001^∗∗^
Mean hemoglobin (g/dL) (SD) (13.5–17.5)	11.9 (±2.2)	12.2 (±2.2)	0.026^∗∗^
Mean ferritin (ng/mL) (SD) (23.9–336.2)	51 (±86.7)	36 (±59.7)	0.001^∗∗^
Mean transferrin saturation (%) (SD) (15–50)	12.8 (±11.5)	11.9 (10.1)	0.318^∗∗^
Received treatment for anemia (*n*, %)	151 (41.59%)	148 (37.37%)	0.255^∗^
Did not use NSAID (*n*) (%)	48 (13.22%)	8 (2.02%)	<0.001^∗^
Used NSAID (*n*) (%)	315 (86.77%)	388 (97.97%)	
Used steroids (*n*) (%)	258 (71.07%)	193 (48.73%)	<0.001^∗^
Used immunomodulatory agents (*n*) (%)	256 (70.52%)	122 (30.80%)	<0.001^∗^
Used biologic agents (*n*) (%)	107 (29.47%)	28 (7.07%)	<0.001^∗^

^∗^Chi-squared test; ^∗∗^Mann–Whitney *U* test. CrD: Chron's disease; UC: ulcerative colitis; SD: standard deviation; NSAID: nonsteroidal anti-inflammatory drug; g/dL: gram per decilitre; ng/mL: nanogram per millilitre.

**Table 2:** The clinical characteristics of patients with celiac disease and IBD.

Gender/age	IBD type/involvement	IBD duration/time between CD diagnosis and IBD diagnosis (years)	Therapy	Anti-TTG IGA (RU/ML)	Anti-EMA IGA (dilution)	Marsh score	Endoscopic findings
Female/38	CrD/distal colon involvement	9/8	5-ASA, methyl prednisolone, AZA	20.20	1/32-1/100	3c	Bulbus and duodenum 2nd segment appear pale, edematous, atrophic, comb tooth appearance
Female/56	CrD/terminal ileum	11/11	5-ASA	<2	1/10-1/32	3b	Normal appearing duodenum
Female/77	UC/distal colitis	3/1	5-ASA	55.87	1/320-1/1000	2	Duodenum 2nd segment has comb sign
Female/31	CrD/perianal fistula, colon involvement	23/1	5-ASA, methyl prednisolone, azathioprine	125.04	1/320-1/1000	3b	Duodenum 2nd segment has comb tooth appearance

IBD: inflammatory bowel disease; CrD: Crohn's disease; UC: ulcerative colitis; CD: celiac disease; 5-ASA: 5 acetyl salicylic acid; AZA: azathioprine; RU/ML: relative unit/millilitre; anti-TTG: anti-tissue transglutaminase; anti-EMA: antiendomysium.

## Data Availability

The (demographic features, laboratory findings, endoscopic findings, and resolving results) data used to support the findings of this study are available from the corresponding author (Göksel Bengi) upon request.
